# Deafness-related protein PDZD7 forms complex with the C-terminal tail of FCHSD2

**DOI:** 10.1042/BCJ20220147

**Published:** 2022-06-24

**Authors:** Huang Wang, Dange Zhao, Haibo Du, Xiaoyan Zhai, Shaoxuan Wu, Lin Lin, Zhigang Xu, Qing Lu

**Affiliations:** 1 Key Laboratory for the Genetics of Developmental and Neuropsychiatric Disorders, Ministry of Education, Bio-X Institutes, Shanghai Jiao Tong University, Shanghai, China; 2 Shandong Provincial Key Laboratory of Animal Cell and Developmental Biology, School of Life Sciences, Shandong University, Qingdao, Shandong, China; 3 Shandong Provincial Collaborative Innovation Center of Cell Biology, Shandong Normal University, Jinan, Shandong, China; 4 Department of Otolaryngology-Head and Neck Surgery, Shanghai Ninth People's Hospital, Shanghai Jiao Tong University School of Medicine, Shanghai, China; 5 Ear Institute, Shanghai Jiao Tong University School of Medicine, Shanghai, China; 6 Shanghai Key Laboratory of Translational Medicine on Ear and Nose Diseases, Shanghai, China

**Keywords:** ankle link, complex structure, FCHSD2, PDZD7, stereocilia

## Abstract

In cochlea, deafness-related protein PDZD7 is an indispensable component of the ankle link complex, which is critical for the maturation of inner-ear hair cell for sound perception. Ankle links, connecting the different rows of cochlear stereocilia, are essential for the staircase-like development of stereocilia. However, the molecular mechanism of how PDZD7 governs stereociliary development remains unknown. Here, we reported a novel PDZD7-binding partner, FCHSD2, identified by yeast two-hybrid screening. FCHSD2 was reported to be expressed in hair cell, where it co-operated with CDC42 and N-WASP to regulate the formation of cell protrusion. The association between FCHSD2 and PDZD7 was further confirmed in COS-7 cells. More importantly, we solved the complex structure of FCHSD2 tail with PDZD7 PDZ3 domain at 2.0 Å resolution. The crystal structure shows that PDZD7 PDZ3 adopts a typical PDZ domain topology, comprising five β strands and two α helixes. The PDZ-binding motif of FCHSD2 tail stretches through the αB/βB groove of PDZD7 PDZ3. Our study not only uncovers the interaction between FCHSD2 tail and PDZD7 PDZ3 at the atomic level, but also provides clues of connecting the ankle link complex with cytoskeleton dynamics for exploiting the molecular mechanism of stereociliary development.

## Introduction

Hearing is initiated by sound waves, which travel through the auditory canal to exert mechanical forces on stereocilia in the inner ear [1]. Actin-based stereocilia localize on the apical surfaces of inner-ear hair cells. Stereocilia are organized into three rows with increasing heights and manifest as a staircase-like pattern, which is essential for sensing sound waves and the following mechano-transduction [1–3]. There are several types of links bridging stereocilia within and across different rows of stereocilia: tip link, top connector, lateral link, and ankle link [4]. Ankle link is present at the stage of postnatal day (P2) to P12 in the mouse cochlea, which is essential for the staircase-like development of stereocilia [5,6]. Ankle link is anchored to a large protein complex (ankle link complex) in the submembrane region. The ankle link complex, also called USH2 protein complex, is composed of Whirlin, Usherin, PDZD7, and VLGR1 (encoded by *WHRN*, *USH2A*, *PDZD7*, and *ADGRV1*, respectively) [7–11]. All the four proteins are critical for the normal stereociliary development of staircase-like pattern. Moreover, mutations of these genes are associated with deafness [12–16].

PDZD7, the key component of the ankle link complex, contains PDZ1–2 in tandem, a HHD (harmonin homology domain), a proline-rich (PR) region, and a third PDZ domain (PDZ3) (Figure 2A) [17]. PDZ1–2 is reported to be involved in the organization of USH2 complex [7]. Meanwhile, HHD is responsible for membrane targeting [17]. But the function of PDZD7 PDZ3 remains elusive. More importantly, the mechanism underlying PDZD7-mediated stereociliary staircase-like development is also puzzling to us.

Stereocilia are actin-based protrusions on hair cells. Several actin-binding proteins regulating actin turnover were reported to be involved in stereociliary elongation [18–26]. FCHSD2 is expressed in hair cell and localizes along the stereocilia in a punctuate pattern, where it co-operates with CDC42 and N-WASP in regulating apical cell protrusion formation [27,28]. FCHSD2 encompasses a F-BAR domain and two SH3 domains. At the extreme C-terminus of FCHSD2, there is a type I PDZ-binding motif (PBM, a.a. sequence Ile–Thr–Leu–Val) for associating with PDZ-containing protein (Figure 2A) [27,29].

In this study, we uncover the interaction between FCHSD2 and PDZD7 through yeast two-hybrid screening and further verify the interaction using Coimmunoprecipitation (Co-IP) assays. Then we proceed to report the complex structure of FCHSD2 tail with PDZD7 PDZ3 at 2.0 Å resolution. The complex structure shows that PDZD7 PDZ3 is a typical PDZ domain, as it is composed of five β strands and two α helixes. The PBM on FCHSD2 tail stretches through the αB/βB groove to insert into the hydrophobic pocket of PDZD7 PDZ3. The present study not only elucidates the interaction between FCHSD2 tail and PDZD7 PDZ3 at the atomic level, but also provides clues for establishing the association between ankle link complex and cytoskeleton dynamics for exploiting the molecular mechanism of stereociliary development.

## Results

### FCHSD2 identified as a PDZD7-binding partner

**Figure 1.**
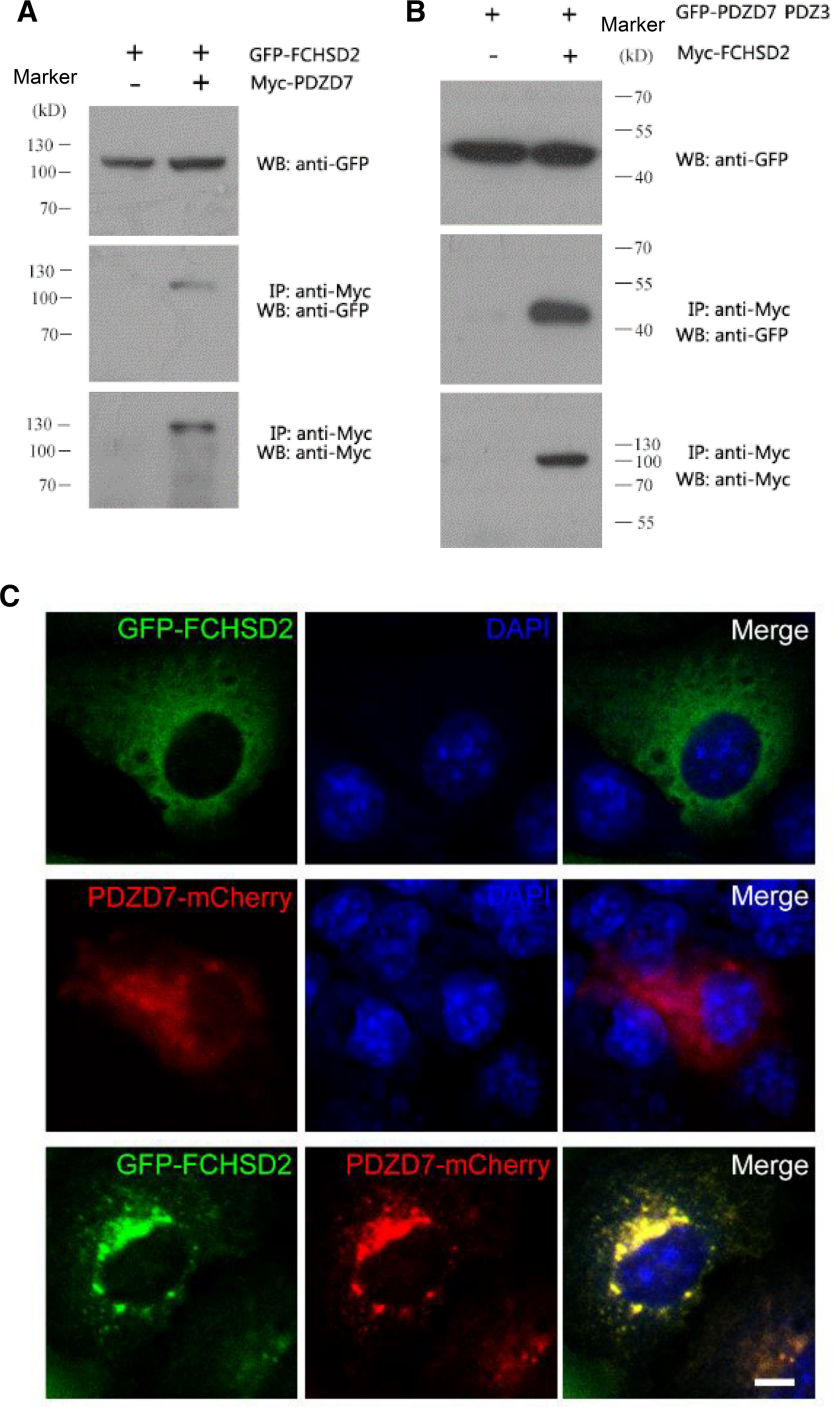
FCHSD2 is a PDZD7-binding partner. (**A**,**B**) Western blots showing that (**A**) GFP-FCHSD2 is co-IPed with Myc-PDZD7, and (**B**) GFP-PDZD7 PDZ3 is co-IPed with Myc-FCHSD2. Expression vectors were transfected into HEK293T cells to express epitope-tagged proteins, and cell lysates were subjected to immunoprecipitation. IP indicates antibody used for immunoprecipitation, and WB indicates antibody used for detection. (**C**) Expression vectors were transfected into COS-7 cells and the subcellular localization of target proteins was determined using confocal microscopy. Scale bar, 20 μm.

Deafness-related protein PDZD7 is critical for the normal staircase-like development of stereocilia [14]. However, the molecular mechanism of PDZD7 governing stereociliary development remains unknown. Thus, yeast two-hybrid screening was performed to identify PDZD7-binding partners. Actually, the N-terminal fragment of PDZD7 (PDZ1–2) has been taken advantage as the bait in our previous study, but no actin-binding protein was found among the binding partners [30]. Thus the C-terminal fragment of mouse PDZD7 (a.a. 559–1021) was used in screening a chicken cochlear cDNA library this time. This fragment includes the HHD, the PR region, and the third PDZ domain (PDZ3). Thirty-seven positive clones were obtained that encode four proteins. Among the identified potential PDZD7-binding partners, FCHSD2 attracted our attention, as we previously have shown that FCHSD2 is a stereociliary protein, which co-operates with CDC42 and N-WASP to regulate F-actin polymerization as well as cell protrusion formation [28]. Co-IP experiments confirmed the interaction between FCHSD2 and PDZD7 in a physiologically more relevant context in transfected HEK239T cells (Figure 1A). The last four amino acids of FCHSD2 constitute a type I PDZ-binding motif (PBM) for PDZ recognition [29], and consistently our Co-IP result showed that PDZD7 PDZ3 (a.a. 842–946) was responsible for the interaction with FCHSD2 (Figure 1B). Furthermore, when overexpressed alone in COS-7 cells, both FCHSD2 and PDZD7 were localized diffusely in the cytoplasm (Figure 1C). However, when expressed together, they colocalized in the perinuclei region in puncta (Figure 1C). Taken together, our present data suggest that FCHSD2 is a novel PDZD7-binding partner, which provides a clue for elucidating PDZD7-mediated control of stereociliary development.

### FCHSD2 tail specifically binds to PDZD7 PDZ3

**Figure 2.**
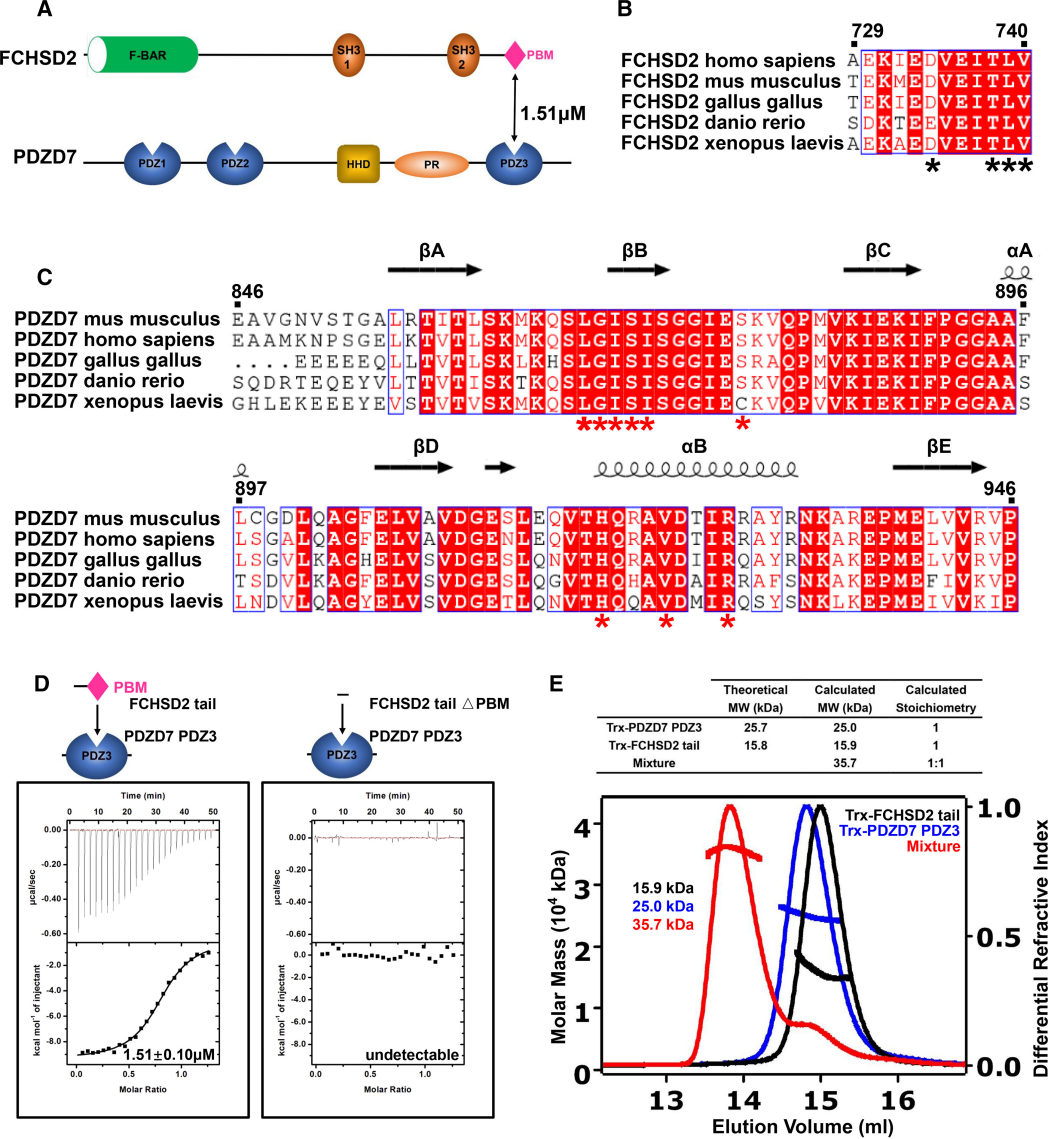
FCHSD2 tail specifically binds to PDZD7 PDZ3. (**A**) Schematic diagram showing the domain organizations of FCHSD2 and PDZD7. (**B**,**C**) Sequence alignments showing that FCHSD2 tail and PDZD7 PDZ3 are highly conserved among different species. The secondary structural elements of PDZD7 PDZ3 determined from this work are shown above its alignment. In these alignments, invariant residues are highlighted with red boxes, the conserved residues are colored in red. Specifically, residues indicated with black or red asterisk are responsible for the interaction between FCHSD2 tail and PDZD7 PDZ3. (**D**) ITC analysis showing that FCHSD2 tail binds to PDZD7 PDZ3 with a *K*_d_ value of ∼1.51 μM. △PBM abolishes FCHSD2 tail's binding to PDZD7 PDZ3. (**E**) SEC-MALS analysis showing that Trx-tagged FCHSD2 tail binds to Trx-tagged PDZD7 PDZ3 at the stoichiometry of 1 : 1. △PBM, the truncation of PBM; PR, proline rich; HHD, harmonin homology domain; PBM, PDZ-binding motif; Trx, thioredoxin.

To uncover the detailed molecular mechanism of FCHSD2/PDZD7 interaction, we generated the FCHSD2 tail construct (a.a. 729–740). Isothermal titration calorimetry (ITC)-based assay showed that FCHSD2 tail bound to PDZD7 PDZ3 with a *K*_d_ value of ∼1.51 μM (Figure 2D, left panel, summarized in Figure 4B). However, FCHSD2 tail displayed no binding to PDZ1 or PDZ2 of PDZD7 (Supplementary Figure S1b1 and b2). In stereocilia, deafness-related protein Whirlin is a paralog of PDZD7 [14]. Whirlin also contains three PDZ domains: PDZ1, PDZ2, and PDZ3 (Supplementary Figure S1A). Among the three PDZ domains, PDZ1 and PDZ2 form a supramodule, enabling fine-tuning of affinities for its binding partners [31]. Additionally, N-terminal domain (NTD) may also form a supramodule with PDZ1, mirroring Whirlin's paralog Harmonin [32]. Thus we generated the NPDZ12 construct, which included NTD, PDZ1, and PDZ2. ITC assays showed that FCHSD2 tail did not bind to NPDZ12 or PDZ3 of Whirlin (Supplementary Figure S1c1 and c2). These results indicate the selectivity of FCHSD2 tail towards the PDZ3 domain of PDZD7.

**Figure 3.**
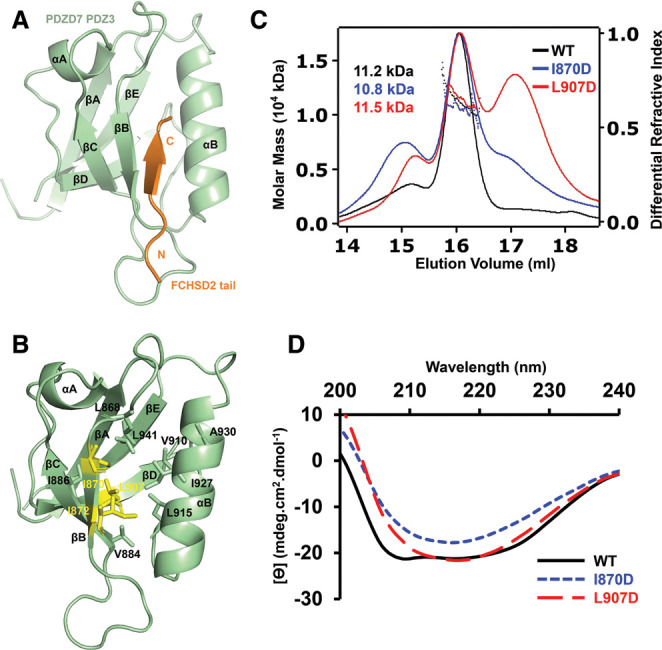
The structure of the FCHSD2 tail/PDZD7 PDZ3 complex. (**A**) Overall structure of the FCHSD2 tail/PDZD7 PDZ3 complex. FCHSD2 tail and PDZD7 PDZ3 are colored in orange and green, respectively. (**B**) Residues responsible for the formation of the hydrophobic core of PDZD7 PDZ3 are shown with stick model. PDZD7 PDZ3 is colored in green. Residues Ile870, Ile872, and Leu907 taken for mutation are colored in yellow. (**C**) SEC-MALS analysis showing that I870D and L907D compromise the homogeneity of PDZD7 PDZ3. (**D**) CD spectrum showing that I870D and L907D disrupt the normal PDZ folding of PDZD7 PDZ3.

**Figure 4.**
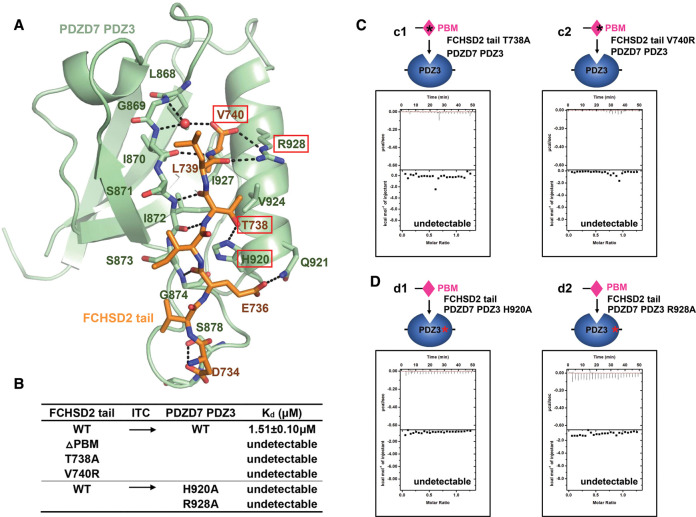
Detailed interactions between FCHSD2 tail and PDZD7 PDZ3. (**A**) Detailed interactions between FCHSD2 tail and PDZD7 PDZ3. Residues involved in protein interaction are shown with stick model. FCHSD2 tail and PDZD7 PDZ3 are colored in orange and green, respectively. Residues taken for mutations are highlighted in red boxes. Hydrogen bonds are shown as black dashed lines. (**B**) Summary of the binding affinities between WT or structural mutants of FCHSD2 tail and PDZD7 PDZ3. (**C**) ITC assays showing that mutant (c1) T738A or (c2) V740R abolishes FCHSD2 tail's binding to PDZD7 PDZ3. (**D**) ITC assays showing that mutant (d1) H920A or (d2) R928A abolishes PDZD7 PDZ3’ binding to FCHSD2 tail.

The truncation of PBM (△PBM) abolished FCHSD2 tail's binding to PDZD7 PDZ3, suggesting that the interaction was indeed mediated by PDZ/PBM binding (Figure 2D, right panel, summarized in Figure 4B). To further elucidate their binding ratio, we tagged both proteins with thioredoxin (Trx) tag and resorted them to size exclusion chromatography with multi-angle light scattering (SEC-MALS) to calculate the relative molecular masses and fit the stoichiometry. It was found that both Trx-tagged FCHSD2 tail and Trx-tagged PDZD7 PDZ3 were stable monomer in solution, as their relative molecular masses were calculated to be ∼15.9 and 25.0 kDa, respectively, which were comparable to their theoretical molecular mass. When they were mixed, the relative molecular mass of the mixture was calculated to be ∼35.7 kDa, suggesting that the two proteins formed a stable complex in solution. The stoichiometry was fitted to be 1 :1, indicating that each molecule of the protein complex is composed of one molecule of FCHSD2 tail and one molecule of PDZD7 PDZ3 (Figure 2E).

### FCHSD2 tail/PDZD7 PDZ3 protein complex adopts a canonical PDZ/PBM binding mode

We proceed to solve the complex crystal structure of FCHSD2 tail/PDZD7 PDZ3 by the molecular replacement method at the resolution of 2.0 Å. Each asymmetric unit contains two complex molecules. Consistent with our biochemical characterization, the FCHSD2 tail/PDZD7 PDZ3 complex shows a 1 : 1 stoichiometry in the crystal structure. Through the structure, it is observed that PDZD7 PDZ3 adopts a canonical PDZ domain architecture. Five antiparallel β strands (βA–βE) form an open β-barrel, which is packed with two α-helices (αA and αB). Typically, the groove formed by αB and βB serves as the docking site for the ligand. FCHSD2 tail stretches along the groove in antiparallel with βB and inserts into the hydrophobic pocket of PDZD7 PDZ3 (Figure 3A).

To investigate the effects of the hydrophobic-interaction network, Ile870, Ile872, or Leu907 were mutated to Asp to disrupt the hydrophobic interactions in the folding core of PDZD7 PDZ3 (Figure 3B). PDZD7 PDZ3 became insoluble when the mutation I872D was introduced (Supplementary Figure S2). Protein homogeneity of PDZD7 PDZ3 was compromised by I870D and L907D (Figure 3C). Circular dichroism (CD) spectrum indicated a well-folded WT protein containing both α-helix and β-strand. In sharp contrast, the spectra of mutants I870D and L907D manifested in a distinctly different pattern, indicating that the two mutant proteins were structurally unfolded (Figure 3D). These results help us draw the conclusion that the internal hydrophobic interactions are critical for the folding of the PDZD7 PDZ3.

### The detail of the interaction between FCHSD2 tail and PDZD7 PDZ3

FCHSD2 tail forms the complex with PDZD7 PDZ3 that buries a total interface area of 526.6 Å^2^. The ligand-binding groove on PDZD7 PDZ3 is formed by the carboxylate-binding loop, αB, and βB. The binding affinity of the FCHSD2/PDZD7 interaction is achieved through networks of hydrophobic interactions and hydrogen bonds. Val740 from FCHSD2 tail inserts into the hydrophobic pocket of PDZD7 PDZ3 to form hydrophobic interactions with Leu868, Ile870, Ile872, and Val924 of PDZD7. In addition to hydrophobic interactions, residues from the carboxylate-binding loop, αB, and βB make hydrogen bonds with FCHSD2 tail. The main chain atoms of Ile870, Ile872, and Gly874 from the βB form hydrogen bonds with Val740, Thr738, and Glu736 from FCHSD2, and stabilize the interaction (Figure 4A).

In a typical PDZ/PBM binding mode, the main chain amides of the Gly–Leu–Gly–Phe motif of the carboxylate-binding loop form hydrogen bonds with the terminal carboxylate group directly. Intriguingly, in the FCHSD2/PDZD7 structure, Gly869 and Ile870 at the carboxylate-binding loop provide amide nitrogens to interact with the carbonyl oxygens of Val740 via a highly ordered water molecule. Arg928 also participates in interactions with the carboxylate to further stabilize the terminal carboxylate group. There are some other hydrogen bonds contributing to the FCHSD2 tail/PDZD7 PDZ3 binding affinity. There are hydrogen bonds between the carbonyl oxygens of Leu739, and the side chain of Arg928. There are also hydrogen bonds between the side chain of Thr738 and the imidazole ring of His920, between the carbonyl oxygen of Glu736 and the side chain of Gln921, between the amide nitrogen of Asp734 and the side chain of Ser878. All the residues involved in the interaction are invariant or highly conserved throughout evolution (Figure 2B,C).

Based on these detailed structural analyses above, we designed mutations to disrupt the interaction between FCHSD2 tail and PDZD7 PDZ3. ITC assays were used to measure the binding affinities and evaluate these critical residues responsible for the interaction. For FCHSD2 tail, Thr738 was mutated to Ala to disrupt the hydrogen bonds, whereas hydrophobic Val740 was mutated to hydrophilic Arg to disrupt hydrogen bonds and hydrophobic interactions. For PDZD7 PDZ3, His920 and Arg928 were mutated to Ala to disrupt the hydrogen bonds. All the designed mutations abolished the interaction between FCHSD2 tail and PDZD7 PDZ3, indicating their critical roles in the FCHSD2 tail/PDZD7 PDZ3 binding (Figure 4c1 and c2, d1 and d2, summarized in Figure 4B).

### The colocalization of FCHSD2 and PDZD7 in cells

**Figure 5.**
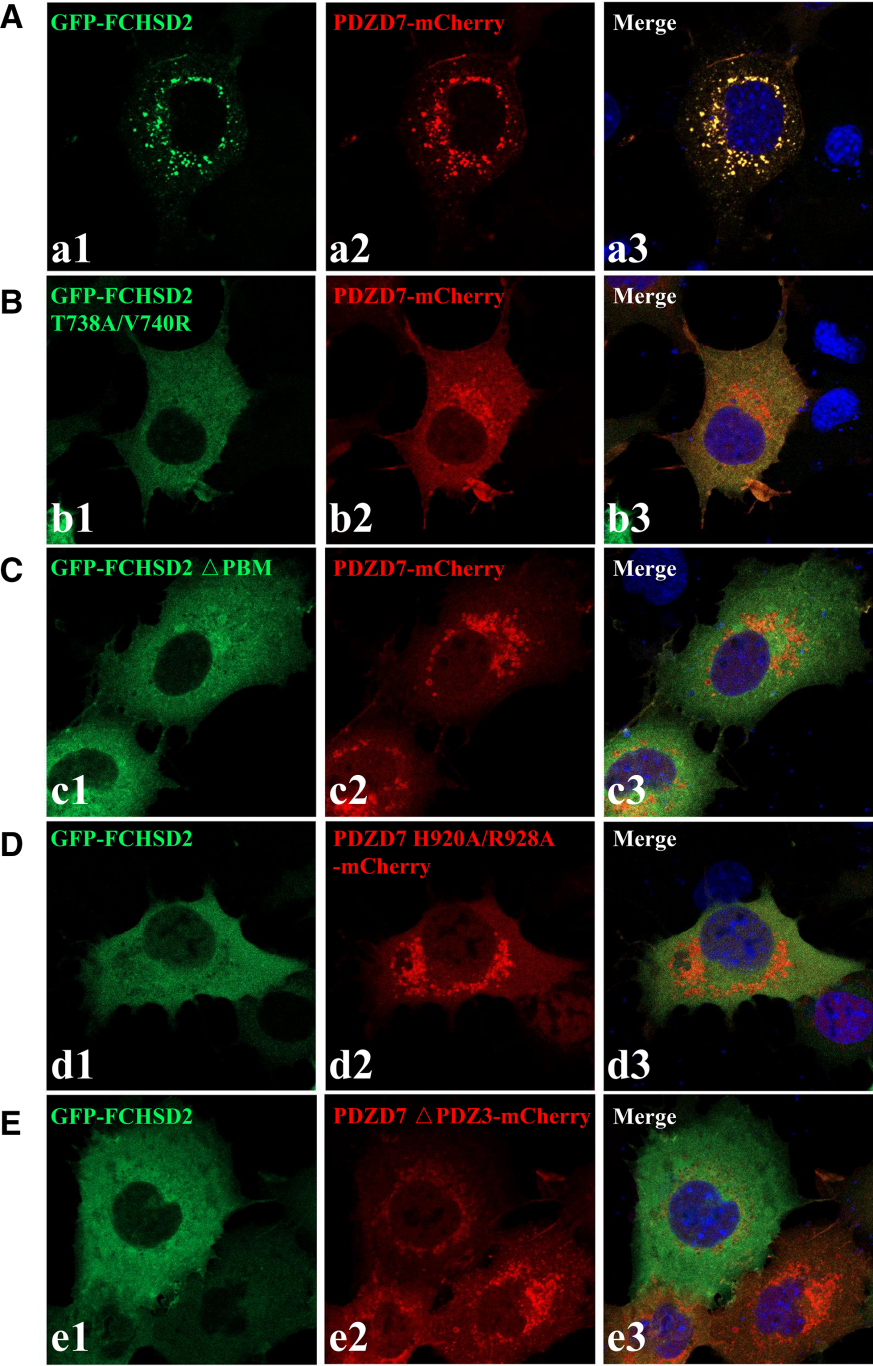
Mutations disrupt the cell colocalization of FCHSD2 and PDZD7. (**A**) GFP-FCHSD2 and PDZD7-mCherry colocalize in the cytoplasm of COS-7 cells in a punctuate pattern. (**B**–**E**) T738A/V740R (**B**) or △PBM (**C**) disrupts GFP-FCHSD2's colocalization with PDZD7-mCherry; H920A/R928A (**D**) or △PDZ3 (**E**) disrupts PDZD7-mCherry's colocalization with GFP-FCHSD2. Scale bar, 20 μm. △PBM or △PDZ3, the truncation of PBM or PDZ3; PBM, PDZ-binding motif.

Both FCHSD2 and PDZD7 have been observed to localize in stereocilia. FCHSD2 mainly localizes along the stereocilia in a punctuate pattern, and PDZD7 is enriched at the ankle link region of the stereocilia [14,27]. We hypothesized that there was a potential interaction between FCHSD2 and PDZD7 in stereocilia. To test this hypothesis, we took advantage of the COS-7 cells to make the investigation. As discovered in Figure 1C, when expressed together, FCHSD2 and PDZD7 colocalized in the perinuclei region in punctuate pattern (Figure 5A). As expected, the two proteins failed to colocalize with each other when the PBM on FCHSD2 or PDZ3 on PDZD7 was deleted, indicating that the colocalization was mediated by the binding between the PBM on FCHSD2 tail and PDZ3 on PDZD7 (Figure 5C,E). To make a further investigation of the responsible amino acid residues for the colocalization on the two proteins, we introduced double mutations into FCHSD2 or PDZD7. Based on our ITC results, we selected T738A/V740R or H920A/R928A for FCHSD2 or PDZD7, respectively. Nicely consistent with the ITC assays, we found that there was no colocalization of the two proteins when either of the double-mutant proteins was adopted in the cell assay (Figure 5B,D). Based on the results from our cell assay, we propose that the colocalization between the two proteins was mediated by the PDZ/PBM binding.

## Discussion

Ankle links are present between different rows of stereocilia at the stage of P2 to P12 and are essential for their staircase-like development. There is a protein complex helping anchor ankle links to the submembrane region of stereocilia. This ankle link complex, also called USH2 complex, is composed of Whirlin, Usherin, VLGR1, and PDZD7 [7–11]. All the four proteins are critical for the normal stereociliary development of staircase-like pattern. Moreover, they are associated with hearing loss [12–16]. However, the molecular mechanism of how the ankle link complex governs stereociliary development remains unknown. The present study provides an important clue by uncovering the interaction between FCHSD2 tail and PDZD7 PDZ3. The association was confirmed by investigation using COS-7 cells. It has been previously discovered that FCHSD2 co-operates with CDC42 and N-WASP in regulating apical cell protrusion formation [27,28]. Thereby the association is established between the ankle link complex and cytoskeleton dynamics for exploiting the molecular mechanism of stereociliary development.

It is worth noting that both PDZD7 and FCHSD2 are capable of being associated with the membrane: PDZD7 binds to the membrane via its HHD [17], while FCHSD2 targets to the membrane with its F-BAR domain [33]. The membrane-targeting abilities of both proteins will enhance the association of PDZD7 with FCHSD2 at the submembrane region and further strengthern the anchor of the cytoskeleton to the plasma membrane.

PDZ domain is a versatile protein–protein interacting module [34]. PDZ domains are classified into three types (I, II, and III) according to specificity for C-terminal peptides, which is chiefly determined by the residues at the 0 and −2 positions of the peptide ligands. Type I PDZ domain specifically recognizes C-terminal peptide with the consensus sequence of X-S/T-X-V, or X-S/T-X-L, where X stands for any unspecified amino acid [35]. Our knowledge of PDZ domain originates from PSD-95. 5HT2C receptor is the binding partner of PSD-95 PDZ1 [36]. It contains type I PBM at its C-terminal end, with the amino acid sequence of Ile–Ser–Ser–Val.

Type I PDZ/PBM binding has been found to play important roles in the organization of protein complex in stereocilia. For instance, Myo15 PBM, with the C-terminal sequence of Ile–Thr–Ile–Leu, just belongs to type I PBM. The interaction between Myo15 PBM and Whirlin PDZ3 has been identified. More importantly, the protein complex structure has also been resolved [37].

Superimposing FCHSD2 tail/PDZD7 PDZ3 with 5HT2C receptor/PSD-95 PDZ1 (PDB access code 2HMO) or Myo15 PBM/Whirlin PDZ3 (PDB access code 6KZ1), we find that FCHSD2 tail binds to PDZD7 PDZ3 in the same mode as 5HT2C receptor or Myo15 PBM binds to their interacting PDZ domain (Supplementary Figure S3A and B). The PBM of FCHSD2 tail, 5HT2C receptor, or Myo15 occupies the groove formed by αB and βB of the PDZ domain to get contacts. Specifically, L(0) and T(−2) from Myo15 PBM mediate its interaction with Whirlin PDZ3 [37]. For the PBM of FCHSD2 tail, the amino acid residues at the 0 and −2 positions are Val740 and Thr738, respectively. The binding between FCHSD2 tail and PDZD7 PDZ3 is totally abolished when the V(0) and T(−2) are mutated to Ala and Arg, respectively, indicating that they are indispensable for the interaction. It is worth noting again that although FCHSD2 tail/PDZD7 PDZ3 shares the same binding mode with Myo15 PBM/Whirlin PDZ3, differences in the critical amino acid residues do not allow the interaction between FCHSD2 tail and Whirlin PDZ3, which will be elucidated in the next paragraph. Therefore, the identification of FCHSD2 tail as the interacting partner of PDZD7 PDZ3 and the further resolving of the complex structure extend our knowledge of the role of canonical type I PDZ/PBM binding play in assembling protein complex in stereocilia.

In stereocilia, Whirlin is a paralog of PDZD7 [14]. Both the two proteins contain three individual PDZ domains (PDZ1, PDZ2, and PDZ3). FCHSD2 tail specifically binds to PDZD7 PDZ3 but not the other PDZ domains on PDZD7 and Whirlin. Our data provide us further insights into the molecular mechanism underlying the binding selectivity (Figure 2C and Supplementary Figure S1D). Sequence alignment among different species shows that Arg928 in PDZD7 PDZ3 is strictly conserved in evolution. More importantly, as demonstrated by our ITC assay, PDZD7 PDZ3 does not bind to FCHSD2 tail when Arg928 is mutated to Ala. Therefore, Arg928 is essential for the interaction between PDZD7 PDZ3 and FCHSD2 tail. Sequence alignment between different PDZ domains shows that it is Thr, Lys, or Ala at this locus on the other PDZ domains of PDZD7 or Whirlin. The side chain of Thr is too short to provide hydrogen for the formation of hydrogen bonds with Leu739 or Val740. Though the side chain of Lys is much longer so that it may interact with Val740, it is still not long enough to form hydrogen bond with Leu739. The strictly conserved Gln921 on PDZD7 PDZ3, which forms hydrogen bond with Glu736 of FCHSD2 tail, also catches our attention. For the other PDZ domains, it is Gly, Ser, Ala, Asp, or Arg at this locus. The side chain of Ser is too short to provide the hydrogen required for hydrogen bond formation. Asp is negatively charged, so it will make like-charge repelling with the negatively charged Glu736 of FCHSD2 tail. On Whirlin PDZ3, it is Arg at this locus, which may form salt bridge with Glu736 on FCHSD2 tail. However, as mentioned above, Whirlin PDZ3 lacks the suitable amino acid residue to form hydrogen bond with Leu739 or Val740. Therefore, the differences in the two critical amino acid residues account for the specificity of FCHSD2 tail to PDZD7 PDZ3 but not the other PDZ domains on PDZD7 or Whirlin.

## Materials and methods

### Yeast two-hybrid screen

The yeast two-hybrid screen was performed as previously described [30]. Briefly, yeast strain AH109 (Clontech) was sequentially transformed with the bait plasmid expressing the C-terminal part of mouse PDZD7 (a.a. 559–1021) and a chicken cochlear cDNA library in the HybriZAP two-hybrid vector [38]. A total of 2.95 × 10^7^ transformants were screened using *HIS3* as the primary reporter gene in the presence of 2.5 mM of 3-amino-1,2,4-triazole (3-AT). Further examination using two other reporter genes *ADE2* and *lacZ*, the prey vectors in triple-positive yeast colonies were recovered, and the sequence of cDNA inserts was determined using Sanger sequencing.

### Coimmunoprecipitation and western blot

HEK293T cells were cultured in the Dulbecco's modified Eagle's medium (DMEM) supplemented with 10% heat-inactivated fetal bovine serum (FBS) and 1% penicillin/streptomycin at 37°C in a 5% CO_2_ humidified atmosphere. Plasmids that express EGFP- or Myc-fusion proteins were transfected into cultured cells using LipoMax transfection reagents (Sudgen, Cat. No. 32012). Twenty-four hours after transfection, cells were lysed in ice-cold lysis buffer consisting of 150 mM NaCl, 50 mM Tris at pH 7.5, 1% (vol/vol) Triton X-100, 1 mM PMSF, and 1× protease inhibitor cocktail (Sigma, Cat. No. S8830). After centrifugation at 4°C, the supernatant was incubated with immobilized anti-Myc antibody (Sigma–Aldrich, Cat. No. E6654) at 4°C for 2 h. Immunoprecipitated proteins were then separated by sodium dodecyl sulfate–polyacrylamide gel electrophoresis (SDS–PAGE) and transferred to polyvinylidene difluoride (PVDF) membrane. After blocking in phosphate buffered saline (PBS) containing 5% bovine serum albumin (BSA) and 0.1% Tween-20, the membrane was incubated with anti-Myc (Sigma–Aldrich, Cat. No. M4439) or anti-GFP (Abmart, Cat. No. M20004) antibody at 4°C overnight, followed by incubation with HRP-conjugated secondary antibody (Bio-Rad, Cat. No. 170-6516) at room temperature for an hour. The signals were detected with the enhanced chemiluminescence (ECL) system (Thermo Fisher Scientific).

### Colocalization assay

COS-7 cells were grown on Gelatin-coated glass cover slips and transfected with plasmids that express target WT or mutant proteins fused to EGFP or mCherry as described above. Twenty-four hours after transfection, cells were fixed with 4% paraformaldehyde (PFA) in PBS for 15 min. To visualize the nuclei, cells were incubated with DAPI (Gen-View Scientific Inc., Cat. No. 28718-90-3) for 15 min. The cells were mounted in Glycerol/PBS (1 : 1), and imaged using a confocal microscope (LSM 700, Zeiss).

### Constructs, protein expression, and protein purification

Different constructs were cloned using standard PCR (Vazyme, Nanjing, China) and homologous recombination (Yeasen, Shanghai, China). FCHSD2 tail (UniProtKB: Q3USJ8, a.a. 729–740) was inserted into pET.32M.3C. For PDZD7 (UniProtKB: E9Q9W7), PDZ1 (a.a. 81–167), PDZ2 (a.a. 202–319), and PDZ3 (a.a. 842–946) were inserted into pET.32M.3C or pET.M.3C. For Whirlin (UniProtKB: Q9P202), NPDZ12 (a.a. 33–377, including NTD, PDZ1, and PDZ2), and PDZ3 (a.a. 816–907) were inserted into pET.32M.3C. All the point mutations of FCHSD2 tail and PDZD7 PDZ3 described in this study were created using the PCR-based mutagenesis method. Both primers and DNA sequencing were provided by GENEWIZ (Suzhou, China).

Recombinant proteins were expressed in *Escherichia coli* BL21 (DE3) cells in LB medium at 16°C. Protein purifications were conducted using a nickel-NTA affinity column followed by size-exclusion (SEC) chromatography in the final buffer containing 50 mM Tris–HCl, 1 mM DTT, 1 mM EDTA, PH7.8, and 100 mM NaCl. When it was needed, the tags were cut off from recombinant proteins by adding 3C protease and removed by another step of SEC chromatography.

### Crystallization, data collection, and structure determination

The purified FCHSD2 tail/PDZD7 PDZ3 protein complex was cut off tag and concentrated to ∼13 mg/ml. The crystals were obtained at 16°C by the sitting drop vapor diffusion method, in 0.05 M zinc acetate dehydrate and 20% w/v polyethylene glycol 3350. Crystals were cryoprotected in reservoir solution with 25% glycerol. Diffraction data were collected at BL19U1 at Shanghai Synchrotron Radiation Facility (SSRF, Shanghai, China) and were processed with XDS.

The complex structure was solved by the molecular replacement method with the Whirlin PDZ3 (PDB code: 1UFX) as the searching model by PHASER [39]. Coot and REFMAC were used to refine the structure [40]. The final refinement statistics of the structure were listed in Table 1. Structural diagrams were prepared by PyMOL (http://www.pymol.org).

**Table 1.** **Data collection and refinement statistics**.

*Data collection and processing*	
Crystal	PDZD7-FCSHD2
Source	SSRF-BL19U1
Wavelength (Å)	0.97846
Space group	P2_1_
Unit cell (a, b, c, Å)	41.6, 62.9, 46.0
Unit cell (α, β, γ, °)	90, 95.2, 90
Resolution range (Å)	50–2.00 (2.06–2.00)
No. of unique reflections	15 785 (1120)
Redundancy	6.7 (6.7)
*I*/*σ*(*I*)	10.0 (3.0)
Completeness (%)	98.6 (95.2)
*R*_merge_ (%)^1^	13.6 (77.4)
CC1/2	99.7 (91.5)
*Structure refinement*	
Resolution (Å)	19.68–2.00
*R*_work_^2^/*R*_free_^3^ (%)	20.88/23.70
rmsd bonds (Å)/angles (°)	0.010/1.280
Number of reflections	
Working set	14 172
Test set	1577
Number of protein atoms	1564
Number of solvent atoms	132
Average B factor (Å^2^) (Protein/solvent)	23.2/32.0
Ramachandran plot (%)	
Most favored regions	100
Additionally allowed	0
Outlier	0

Numbers in parentheses represent the value for the highest resolution shell.

1 *R*_merge_ = ∑|*I*_i_ − *I*_m_|/∑*I*_i_, where *I*_i_ is the intensity of the measured reflection and *I*_m_ is the mean intensity of all symmetry-related reflections;

2 *R*_work_ = Σ||*F*_obs_| − |*F*_calc_||/Σ|*F*_obs_|, where *F*_obs_ and *F*_calc_ are observed and calculated structure factors;

3 *R*_free_ = Σ*T*||*F*_obs_| − |*F*_calc_||/Σ*T*|*F*_obs_|, where *T* is a test data set of about 10% of the total reflections randomly chosen and set aside prior to refinement.

### Isothermal titration calorimetry assay

The measurements were carried out on a MicroCal iTC200 system (Malvern Panalytical) at 25°C. Approximately 500 μM proteins were loaded into the syringe and titrated into the cell filled with ∼50 μM potential binding partners. Each titration point was acquired by injecting a 1-μl aliquot of the protein sample from the syringe into the protein sample in the cell. Titration data were analyzed using Origin 7.0 and fitted by a one-site binding model.

### SEC coupled with multi-angle static light scattering

A superose 12 10/300 GL column (GE Healthcare), a multi-angle static light scattering detector (miniDAWN, Wyatt), and a differential refractive index detector (Optilab, Wyatt) were coupled with the AKTA FPLC system. The column was pre-equilibrated overnight. Purified proteins were prepared at ∼300 μl and 50 μM for loading. Data were analyzed using ASTRA 6 (Wyatt).

### Circular dichroism

Proteins were prepared in a quartz cuvette with a 1 mm light path length. CD measurements were conducted with a thermostated cell holder in a J-1500 spectropolarimeter (Jasco, Tokyo). Far-UV spectra in the window of 200–240 nm were recorded at different temperatures (20–80°C). The spectrum of the buffer was taken as baseline. The data recorded at 30°C was plotted using Excel (Microsoft).

## Data Availability

The atomic co-ordinate of FCHSD2/PDZD7 complex has been deposited in the Protein Data Bank under the accession code 7WEG.

## Abbreviations

3-AT: 3-amino-1,2,4-triazole
BSA: bovine serum albumin
CD: circular dichroism
Co-IP: Coimmunoprecipitation
DMEM: Dulbecco's modified Eagle's medium
ECL: enhanced chemiluminescence
FBS: fetal bovine serum
HHD: harmonin homology domain
ITC: isothermal titration calorimetry
MALS: multi-angle static light scattering
P: postnatal day
PBM: PDZ-binding motif
PBS: phosphate buffered saline
PDVF: polyvinylidene fluoride
PFA: paraformaldehyde
SDS–PAGE: sodium dodecyl sulfate–polyacrylamide gel electrophoresis
SEC: size-exclusion chromatography
USH: Usher syndrome
