# Supplementary material for: Deafness-related protein PDZD7 forms complex with the C-terminal tail of FCHSD2
Source: Biochem J. 2022 Jun 24;479(12):1393–405. doi: 10.1042/BCJ20220147 (PMC9317961; doi:10.1042/BCJ20220147)
Supplement: Supplementary Material [file BCJ-479-1393-s1.pdf]

sFig. 1

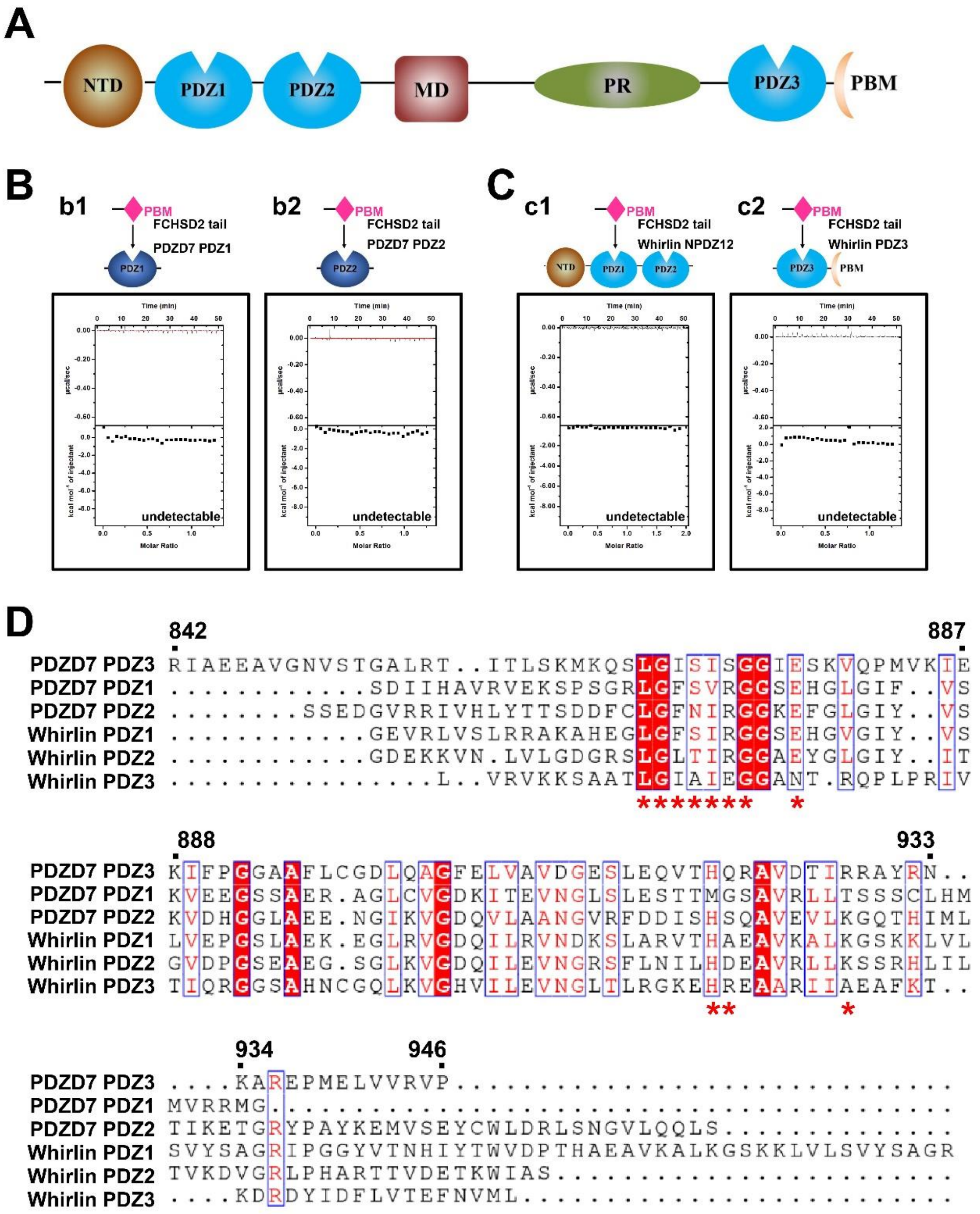
A
NTD
PDZ1
PDZ2
MD
PR
PDZ3
PBM
B
b1
PBM
FCHSD2 tail
PDZD7 PDZ1
PDZ1
b2
PBM
FCHSD2 tail
PDZD7 PDZ2
PDZ2
C
c1
PBM
FCHSD2 tail
Whirlin NPDZ12
NTD
PDZ1
PDZ2
c2
PBM
FCHSD2 tail
Whirlin PDZ3
PDZ3
PBM
Time (min)
µcal/sec
kcal mol-1 of injectant
Molar Ratio
undetectable
D
842
887
PDZD7 PDZ3 RIAEEAVGNVSTGALRT..ITLSKMKQSLGISISGGIESKVQPMVKIE
PDZD7 PDZ1 ............SDIIHAVRVEKSPSGRLGFSVRGGSEHGLGIF..VS
PDZD7 PDZ2 ........SSEDGVRRIVHLYTTSDDFCLGFNIRGGKEFGLGIY..VS
Whirlin PDZ1 ............GEVRLVSLRRAKAHEGLGFSIRGGSEHGVGIY..VS
Whirlin PDZ2 ............GDEKKVN.LVLGDGRSLGLTIRGGAEYGLGIY..IT
Whirlin PDZ3 ................L..VRVKKSAATLGIAIEGGANT.RQPLPRIV
888
933
PDZD7 PDZ3 KIFPGGAAFLCGDLQAGFELVAVDGESLEQVTHQRAVDTIRRAYRN..
PDZD7 PDZ1 KVEEGSSAER.AGLCVGDKITEVNGLSLESTTMGSAVRLLTSSSCLHM
PDZD7 PDZ2 KVDHGGLAEE.NGIKVGDQVLAANGVRFDDISHSQAVEVLKGQTHIML
Whirlin PDZ1 LVEPGSLAEK.EGLRVGDQILRVNDKSLARVTHAEAVKALKGSKKLVL
Whirlin PDZ2 GVDPGSEAEG.SGLKVGDQILEVNGRSFLNILHDEAVRLLKSSRHLIL
Whirlin PDZ3 TIQRGGSAHNCGQLKVGHVILEVNGLTLRGKEHREAARIIAEAFKT..
934
946
PDZD7 PDZ3 ....KAREPMELVVRVP...........................
PDZD7 PDZ1 MVRRMG......................................
PDZD7 PDZ2 TIKETGRYPAYKEMVSEYCWLDRLSNGVLQQLS...........
Whirlin PDZ1 SVYSAGRIPGGYVTNHIYTWVDPTHAEAVKALKGSKKLVLSVYSAGR
Whirlin PDZ2 TVKDVGRLPHARTTVDETKWIAS.....................
Whirlin PDZ3 ....KDRDYIDFLVTEFNVML.......................

sFig. 2

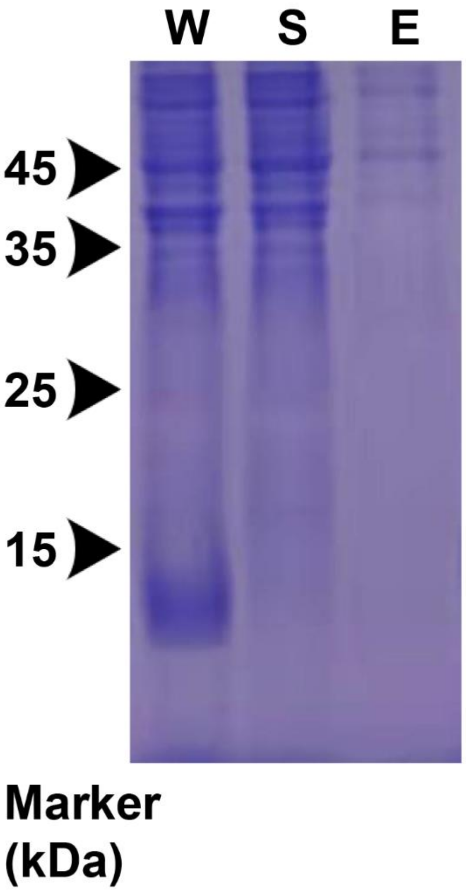

sFig. 3

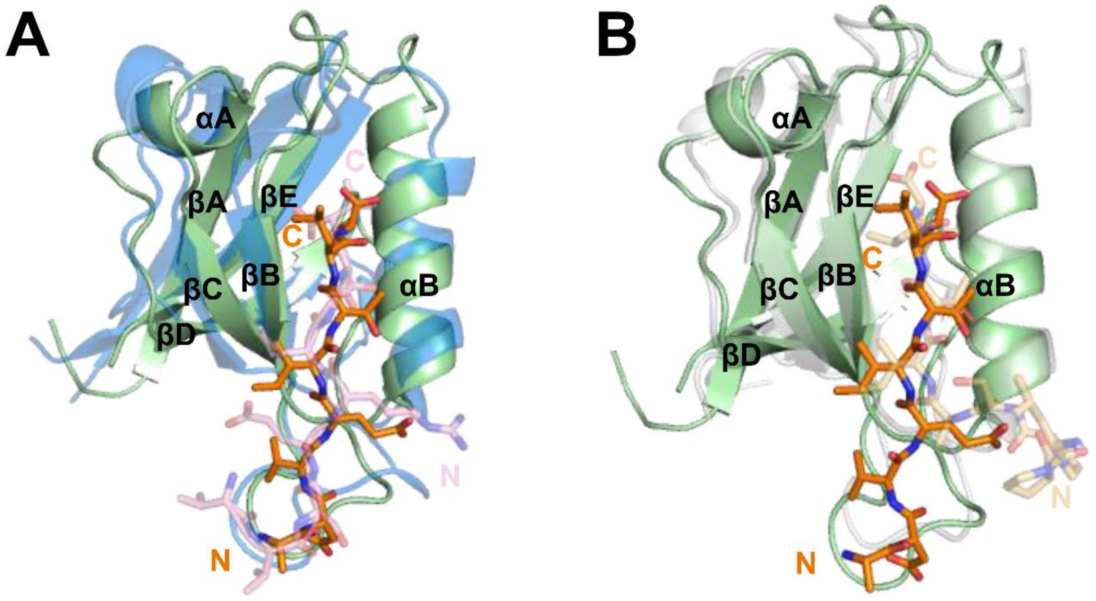

FCHSD2 tail/PDZD7 PDZ3
PSD-95 PDZ1/5HT2C Receptor

FCHSD2 tail/PDZD7 PDZ3
Myo15 PBM/Whirlin PDZ3

Supplementary Figure Legends

sFig. 1. FCHSD2 tail specifically binds to PDZD7 PDZ3 but not the other PDZ domains on PDZD7 and Whirlin.

(A) Schematic diagram showing the domain organizations of Whirlin. (B) ITC assays showing that FCHSD2 tail does not bind to (b1) PDZ1 or (b2) PDZ2 of PDZD7. (C) ITC assays showing that FCHSD2 tail does not bind to (c1) NPDZ12 or (c2) PDZ3 of Whirlin. (D) Sequence alignments between PDZ1, PDZ2, and PDZ3 of both PDZD7 and Whirlin. In these alignments, invariant residues are highlighted with red boxes, the conserved residues are colored in red. Specifically, residues indicated with red asterisk are responsible for the interaction between FCHSD2 tail and PDZD7 PDZ3. The fragment NPDZ12 includes NTD, PDZ1, and PDZ2. NTD, N-terminal domain. MD, middle domain. PR, proline rich. PBM, PDZ binding motif.

sFig. 2. SDS-PAGE analysis showing that PDZD7 PDZ3 become almost insoluble when the mutant I872D is introduced. “W”, “S”, and “E” mean whole-cell extract, supernatant after centrifugation of the cell lysate, and elution from the Ni^{2+}-NTA column, respectively. SDS-PAGE, sodium dodecyl sulfate-polyacrylamide gel electrophoresis.

sFig. 3. Superimposed structural comparison of FCHSD2 tail/PDZD7 PDZ3 (PDB access code 7WEG) with (A) PSD-95 PDZ1/5HT2C Receptor (PDB access code 2MHO) or (B) Myo15 PBM/Whirlin PDZ3 (PDB access code 6KZ1). PBM, PDZ binding motif.
